# A reproducible framework for synthetic data generation and instance segmentation in robotic suturing

**DOI:** 10.1007/s11548-025-03460-8

**Published:** 2025-06-24

**Authors:** Pietro Leoncini, Francesco Marzola, Matteo Pescio, Maura Casadio, Alberto Arezzo, Giulio Dagnino

**Affiliations:** 1 Robotics and Mechatronics, University of Twente, Drienerlolaan 5, 7522 Enschede, NB Netherlands; 2 Department of Surgical Sciences, Università degli Studi di Torino, Corso Dogliotti 14, 10126 Turin, TO Italy; 3 DIMEAS, Politecnico di Torino, Corso Duca degli Abruzzi 24, 10129 Turin, TO Italy; 4 DIBRIS, Università degli Studi di Genova, Via all’Opera Pia 13, 16145 Genoa, GE Italy

**Keywords:** Synthetic data, Simulation, Sim-to-real, Instance segmentation, Data-driven

## Abstract

**Purpose:**

Automating suturing in robotic-assisted surgery offers significant benefits including enhanced precision, reduced operative time, and alleviated surgeon fatigue. Achieving this requires robust computer vision (CV) models. Still, their development is hindered by the scarcity of task-specific datasets and the complexity of acquiring and annotating real surgical data. This work addresses these challenges using a sim-to-real approach to create synthetic datasets and a data-driven methodology for model training and evaluation.

**Methods:**

Existing 3D models of Da Vinci tools were modified and new models–needle and tissue cuts–were created to account for diverse data scenarios, enabling the generation of three synthetic datasets with increasing realism using Unity and the Perception package. These datasets were then employed to train several YOLOv8-m models for object detection to evaluate the generalizability of synthetic-trained models in real scenarios and the impact of dataset realism on model performance. Additionally, a real-time instance segmentation model was developed through a hybrid training strategy combining synthetic and a minimal set of real images.

**Results:**

Synthetic-trained models showed improved performance on real test sets as training dataset realism increased, but realism levels remained insufficient for complete generalization. Instead, the hybrid approach significantly increased performance in real scenarios. Indeed, the hybrid instance segmentation model exhibited real-time capabilities and robust accuracy, achieving the best Dice coefficient (0.92) with minimal dependence on real training data (30–50 images).

**Conclusions:**

This study demonstrates the potential of sim-to-real synthetic datasets to advance robotic suturing automation through a simple and reproducible framework. By sharing 3D models, Unity environments and annotated datasets, this work provides resources for creating additional images, expanding datasets, and enabling fine-tuning or semi-supervised learning. By facilitating further exploration, this work lays a foundation for advancing suturing automation and addressing task-specific dataset scarcity.

## Introduction

Automation in robotic-assisted surgery offers numerous advantages, including enhanced precision, reduced operative times, and alleviation of surgeon fatigue [1]. One of the most promising tasks for automation is suturing—a repetitive, time-consuming, and fundamental component of many surgical procedures [2]. Suturing automation could standardize outcomes, reduce the cognitive burden on surgeons, and improve performance, particularly in robotic-assisted minimally invasive surgery (RAMIS), where ergonomic challenges often impede precision [3, 4].

Despite the evident benefits, automating surgical suturing presents significant challenges. Reliable tool segmentation is a prerequisite for real-time instrument tracking, motion analysis, and subsequent automation [5]. This process relies heavily on cognitive vision systems capable of identifying tools and tissues and performing surgical action recognition with high accuracy [2, 6]. However, developing such systems necessitates large, labeled datasets to train models with millions of parameters. This process is inherently expensive, time-consuming, and error-prone due to the complexity of the surgical environment and the need for specialized annotators [7, 8]. Furthermore, data quality and diversity issues introduce additional hurdles, as biases in real-world datasets often lead to models that fail to generalize effectively to novel or variable contexts [9].

To address these limitations, synthetic data have emerged as a promising alternative [8]. Virtual environments, such as those created using game engines like Unity and Unreal Engine, enable the generation of diverse, annotated datasets with minimal human intervention [10]. These environments allow researchers to simulate surgical scenarios with high variability, effectively mitigating biases and enabling vast data generation at low cost [11]. Moreover, synthetic datasets provide precise control over scene properties, facilitating the creation of task-specific training data, which is particularly scarce for robotic suturing [12]. However, their utility is limited by the significant sim- to-real gap, as models trained solely on synthetic images often struggle to generalize to real-world applications [13].

Recent advancements suggest that combining synthetic and real data can enhance model generalization to real-world scenarios. Blending synthetic datasets with small, well-annotated real datasets can improve performance in out-of-distribution contexts, leveraging the strengths of both data types [11, 14].

Therefore, robotic suturing automation requires effective data solutions and advanced computer vision (CV) models tailored to the task [6]. While most current research relies on binary semantic segmentation, this approach is often insufficient for the precision required in RAMIS [12]. Instance-based segmentation, which also distinguishes between individual objects of the same type, is critical for surgical scene understanding and subsequent tasks such as pose estimation and action recognition—key steps toward suturing automation [5].

This study addresses these challenges by exploring a data-driven methodology prioritizing dataset quality over model architecture optimization. By keeping model hyperparameters constant and systematically varying training datasets, this work evaluates the impact of synthetic and hybrid data on model performance.

This work investigates several key aspects related to the use of synthetic and hybrid data for robotic suturing tasks. Specifically, it explores the generalizability of models trained solely on synthetic datasets to real-world scenarios. It also examines how increasing the realism of synthetic datasets enhances their effectiveness in bridging the sim-to-real gap. Furthermore, the study investigates the influence of incorporating a small number of real images into training datasets on model performance and it seeks to determine the minimum amount of real data required to maintain robust outcomes for robotic suturing tasks.

By addressing these questions, this study seeks to contribute to the advancement of robotic suturing automation. The developed framework offers a reproducible, accessible solution for generating task-specific instance-based datasets, leveraging Unity for synthetic data creation, and combining these with limited real data to minimize annotation efforts. These resources, along with information on data quality and generalization, aim to support the broader research community in overcoming current limitations and advancing automation in robotic-assisted surgery.

## Materials and methods

### Dataset generation

#### 3D modeling

**Fig. 1:**
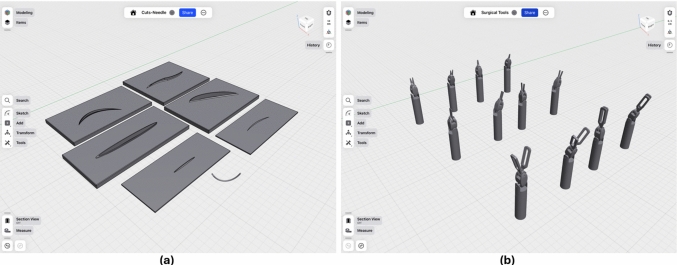
**a** 3D models of cuts and needle created from scratch (**b**) 3D models of Da Vinci tools modified to maintain only the relevant parts. Four states were created for each tool: open, close, folded-open and folded-closed. **b** From top to bottom: Potts Scissors, Needle Driver and Cadiere Forceps

The process of dataset creation for this study involved the design and preparation of task-specific 3D models to simulate realistic surgical scenarios in Unity, representing the endoscope’s point of view. Da Vinci surgical tools, including *cadiere forceps*, *needle drivers* and *potts scissors*, were obtained from the Intuitive GitHub repository.^1^ Using Shapr3D,^2^ these models were modified to retain only the portions of the tools visible in typical endoscopic views, ensuring focus on the critical components relevant to CV tasks (Fig. 1 right). To introduce geometric variability, four distinct states of the tip were developed for each tool: open, closed, folded-closed, and folded-open configurations. This variability was essential to enhance the robustness of models trained on these datasets. In addition, a surgical needle and six distinct tissue cuts were modeled to simulate various suturing elements, all surrounded by a simple pad to replicate a consistent and coherent background (Fig. 1 left).

#### Synthetic data generation: unity

**Fig. 2:**
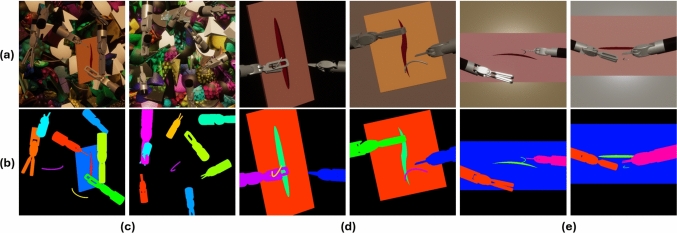
Six representative RGB images (**a**) and their corresponding instance segmentation masks (**b**) from the three synthetic datasets generated. Two arbitrary samples each are shown for Random (**c**), Endoscope1 (**d**), and Endoscope2 (**e**) datasets

**Fig. 3:**
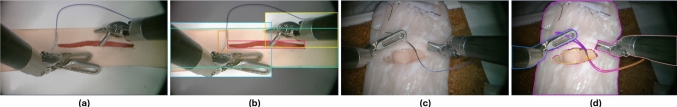
Examples of real RGB images of T1 (**a**) and T2 (c) obtained by sampling videos taken from the Da Vinci endoscope. (**b**) Example of manual annotation of bounding boxes for Yolov8-det on T1 image. (**c**) Example of manual annotation of contours for Yolov8-seg on T2 image

The 3D models were exported in.obj format and subsequently imported into Unity for scene construction. Three distinct scenes were developed to provide varying levels of realism and contextual relevance. The first scene involved random placement of objects, creating a general dataset with random backgrounds and object orientations. This dataset, named *Random*, was designed to ensure that the model could recognize and adapt to various object positions and appearances (Fig. 2). The second scene simulated a typical suturing scenario, where the surgical tools were positioned on either side of the endoscopic field of view with the needle and the tissue positioned centrally (Fig. 2). This dataset is referred to as *Endoscope1*. The third scene was conceptually similar to the second, generating the *Endoscope2* dataset, but incorporated enhanced lighting and rendering techniques to further narrow the gap between simulation and visual context of real-world robotic suturing (Fig. 2), aligning more closely with the realistic scenario encountered in our real-world context (Fig. 3). Using Unity’s Perception package,^3^ a virtual camera was placed within each scene, and tags were assigned to each object. This setup enabled the automatic generation of annotations during simulation, including bounding boxes and instance segmentation masks, for all tagged objects. The simulation process was rendered visible in real time, allowing iterative adjustments to simulation parameters and randomization settings. To enhance variability, C# scripts, called *Randomizers*, were employed to alter object materials, positions, and movements within each scene. Each scenario produced thousands of annotated images (*∼* 5000) in 5–10 s on a laptop (Intel Core i7-8565U CPU, 16 GB RAM). The pipeline demonstrates efficiency and accessibility, enabling researchers with modest computational resources to replicate the process.

#### Real data acquisition and labeling

To create a small dataset of real images, 200 frames were extracted from a video recorded using the endoscope of the da Vinci robotic system, ensuring an evenly spaced selection of representative images (*∼* 1.3 sampling time). The scene depicted Cadiere Forceps and Needle Driver positioned on either side of the endoscopic field of view, with a surgical needle and a colon rubber phantom cut centrally to replicate the real working context (Fig. 3). Due to the absence of *potts scissors*, these are not present in the real dataset. Then, these frames were manually annotated using the Roboflow^4^ platform to generate accurate bounding boxes and segmentation masks for all relevant objects (Fig. 3).

From this annotated dataset, 50 images were used as the first in-distribution test set (T1) to ensure its applicability to real-world robotic suturing scenarios. In addition, to evaluate cross-domain generalization, an out-of-distribution test set (T2) of 50 images was acquired from an ex-vivo scenario using a porcine colon, with varied lighting, background, and camera angles—offering a more realistic testing condition.

### Model training

A *data-driven* approach was employed to evaluate the impact of dataset variations on model performance. Rather than changing the CV model or its hyperparameters, the study kept the same architecture and Ultralytics^5^ default parameter configuration, varying the training datasets to assess their influence on model generalization. As detailed in Table 1, Model 1 was trained solely on the *Random* dataset, Model 2 on a combination of *Random* and *Endoscope1*, Model 3 on *Random*, *Endoscope1*, and *Endoscope2*, and Model 4 incorporated all three synthetic datasets plus a small set of real images. YOLOv8-m was trained for 100 epochs on these four distinct dataset configurations, for both detection (*YOLOv8m-det*) and instance segmentation (*YOLOv8m-seg*) tasks, generating four detection and four instance segmentation models. For each training process, the datasets were partitioned into training and validation sets in proportions of 85% and 15%, respectively. Additionally, 100 images from each dataset were held out for testing, ensuring that these images were not used during training.

**Table 1:** Training-data configuration

	# Random	# Endoscope1	# Endoscope2	# Real	# Total images
Model 1	2500	0	0	0	2500
Model 2	1250	1250	0	0	2500
Model 3	825	825	850	0	2500
Model 4	825	825	850	150	2650

To further investigate the influence of real data on model performance and minimize reliance on real-world data acquisition, a study was conducted to determine the minimum number of real images necessary within the hybrid training set to maintain satisfactory performance. This investigation, performed for object detection and instance segmentation tasks, involved training additional YOLOv8-m models. These training runs maintained a constant number and type of synthetic images while iteratively reducing the number of real images included in the training set. This allowed for a systematic evaluation of the impact of real data quantity on model performance.

### Model evaluation

To comprehensively evaluate the models’ ability to generalize and address the initial research questions posed in this work, four distinct test sets named after each dataset—*Random*, *Endoscope1*, *Endoscope2,* and *Real* —were created. These test sets were designed to assess model performance across diverse scenarios, ranging from synthetic environments with increasing realism to real-world data. Each test set was constructed from the corresponding datasets generated in Unity, ensuring a representative sample of each scenario. Therefore, an *In* and *Out-of-Distribution Testing* process was implemented. This approach distinguished between testing models on images from the same distribution as the training data (in-distribution), though these images were distinct from those used in training, and testing on images from distributions that were never part of the training process (out-of-distribution), to assess performance on images from entirely different scenarios. This dual approach was conducted for both detection and instance segmentation tasks.

Model performance evaluation employed the Dice coefficient and the mean Average Precision (mAP): the Dice coefficient is defined as the harmonic mean of Precision and Recall:

1 $$ Dice = \frac{2TP}{{2TP + FP + FN}} = \frac{2*P*R}{{P + R}} $$

where:

- *TP*: True Positive.
- *FP*: False Positive.
- *FN*: False Negative.
- *P*: Precision.
- *R*: Recall.

The mAP is defined as the average of the areas under the precision-recall curves (AP) computed at different IoU (*IoU* = *TP/(TP* + *FP* + *FN)*) thresholds for a specific object class:

2 $$ mAP_{c} = \frac{1}{N}\mathop \sum \limits_{t = 1}^{N} AP_{c,t} $$

where:

- mAPc: Mean Average Precision for class c.
- *N*: Number of IoU thresholds (t) evaluated.
- APc,t: Average Precision for class c at IoU threshold *t*.

Both metrics range from 0 (poor) to 1 (excellent) and were prioritized in this study for their ability to synthesize Precision and Recall, providing a balanced and comprehensive measure of each model’s performance.

For detection and segmentation tasks, the Dice coefficient quantified the overlap between prediction and corresponding ground truth. This metric offers valuable insights into the accuracy and robustness of the models, emphasizing their ability to align predictions closely with actual object boundaries, offering an overall measure of the model’s performance. Meanwhile, the inclusion of mAP provided additional granularity in the evaluation process, since it enables a deeper analysis of object-specific performance, highlighting disparities in detection or segmentation capabilities across different object categories. Together, these metrics ensured a comprehensive understanding of the models’ effectiveness, capturing strengths and limitations across diverse testing conditions for both detection and segmentation models.

### Framework and tools

The framework for this study was developed using Pytorch (version 2.4.0) and CUDA (version 12.4). YOLOv8-m, a pre-trained CV model developed by Ultralytics (version 8.3.5), was chosen for its demonstrated real-time performance, accuracy and balance offered by its medium-sized version, critical characteristics for surgical applications. Additionally, YOLO has demonstrated excellent performance in real-world surgical scenarios in prior studies [15, 16], further justifying its selection.

All training and evaluation processes were conducted on an NVIDIA GeForce RTX 3070 Ti GPU. 3D models, Unity environments and annotated datasets are made available on GitHub.^6^

## Results

**Fig. 4:**
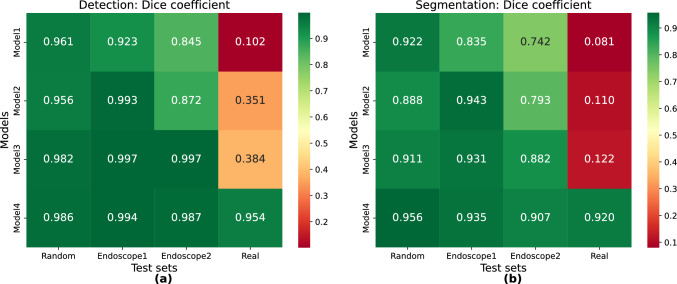
These heatmaps displays the Dice coefficient for each Detection (**a**) and Segmentation (**b**) model across the four different test sets. Higher values indicate a better overlap between predictions and corresponding ground truth, suggesting a lower rate of false positives and false negatives

**Fig. 5:**
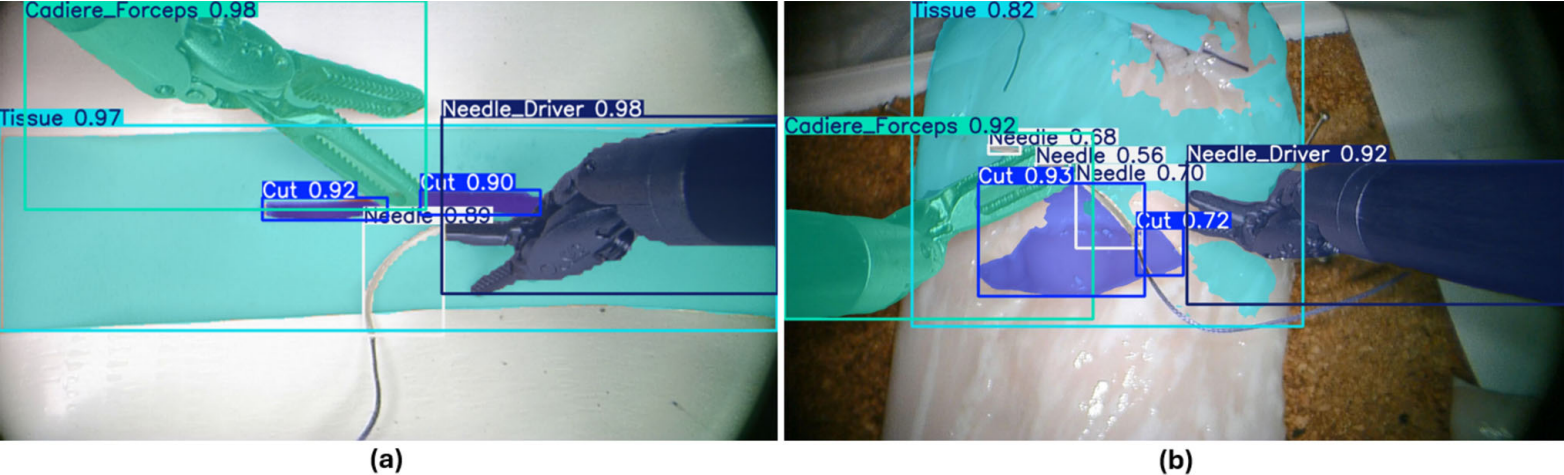
Inference results of the hybrid-trained Model 4 on T1 (**a**) and T2 (**b**). High-performance detection and segmentation of all elements in an in-distribution setting (**a**). In out-of-distribution conditions, performance degrades, leading to challenges in segmenting tissue and cuts (**b**)

Detection tasks demonstrated a clear trend in performance across the four models and test sets (Fig. 4 left). Focusing on Synthetic data, Models 3 and 4 achieved the highest performance metrics in all synthetic test sets (Random, Endoscope1, and Endoscope2), with a Dice ranging between 0.982 and 0.997 for Model 3 and comparable results for Model 4. Considering Real data, the Dice scores increase progressively from Model 1 to Model 4 (0.102, 0.351, 0.384, 0.954), demonstrating the utility of increasing realism in simulated environments to improve model generalization to real scenario. However, Model 4, which incorporated few real images during training, was the only one to perform well in such a scenario (Fig. 5).

Similar trends were observed for segmentation tasks (Fig. 4 right), with a more pronounced performance gap between synthetic and hybrid models in the real scenario. Based on Synthetic data, Models 3 and 4 exhibited superior segmentation performance on synthetic datasets, achieving higher Dice Scores than Models 1 and 2. In addition, Model 4 recorded Dice Scores of 0.967, 0.982, and 0.966 across Random, Endoscope1 and Endoscope2 test sets, respectively, outperforming Model 3, which achieved scores of 0.911, 0.931, and 0.882. This improvement underscores the functionality of a hybrid training strategy, combining synthetic and real images, which enhances performance even in simulated contexts compared to models trained exclusively on synthetic data.

Considering instead Real data, Models 1, 2, and 3 exhibited very low Dice Scores (0.081, 0.110, 0.122). Indeed, unlike detection, the segmentation task—being inherently more complex—did not show an improvement in performance with increasing realism of synthetic datasets alone. However, Model 4, despite using in addition only a minimal number of real images during training, achieved a significantly higher Dice Score of 0.920, demonstrating its effectiveness in bridging the synthetic-to-real gap (Fig. 5). In addition, Model 4 was evaluated on the T2 real test set. Due to the increased domain shift, the model showed reduced performance with a Dice Score of 0.62 for the segmentation and 0.71 for detection.

### Object-specific model performance

**Fig. 6:**
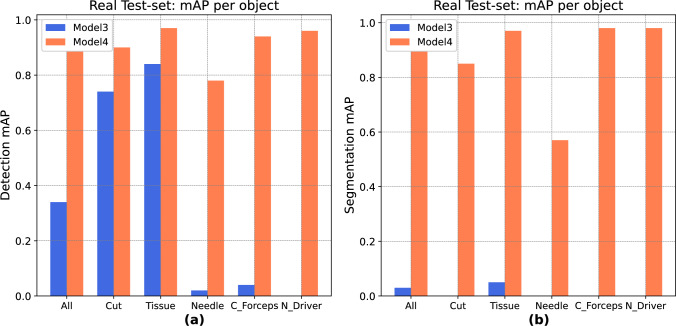
Comparison of mAP performance between Model 3 (trained with synthetic data) and Model 4 (trained with both synthetic and a small set of real data) across Cut, Tissue, Needle, Cadiere Forceps and Needle Driver on the Real test set for both detection (**a**) and segmentation (**b**). Note that Potts Scissors were not present in the Real dataset

To gain insights into the models’ ability to recognize specific objects, mAP was analyzed for both detection and segmentation tasks, considering the real test set (Fig. 6). The segmentation mAP per object reveals a similar pattern to detection, with a higher gap between synthetic and hybrid models.

In the real test set, Model 3 demonstrated limited object recognition capabilities in detection, achieving reasonable mAP only for Cut and Tissue (mAP: 0.74–0.83). At the same time, its performance dropped for Needle, Cadiere Forceps and Needle Driver. In segmentation, Model 3’s mAP was close to zero for all object classes. Model 4, in contrast, achieved significantly higher mAP values across all objects in both detection (mean mAP: 0.9) and segmentation (mean mAP: 0.87) on the real test set, demonstrating superior generalization to real-world scenarios. However, even for Model 4, the needle presented the greatest challenge for segmentation (mAP: 0.57), indicating a potential area for further improvement.

### Real data impact

**Fig. 7:**
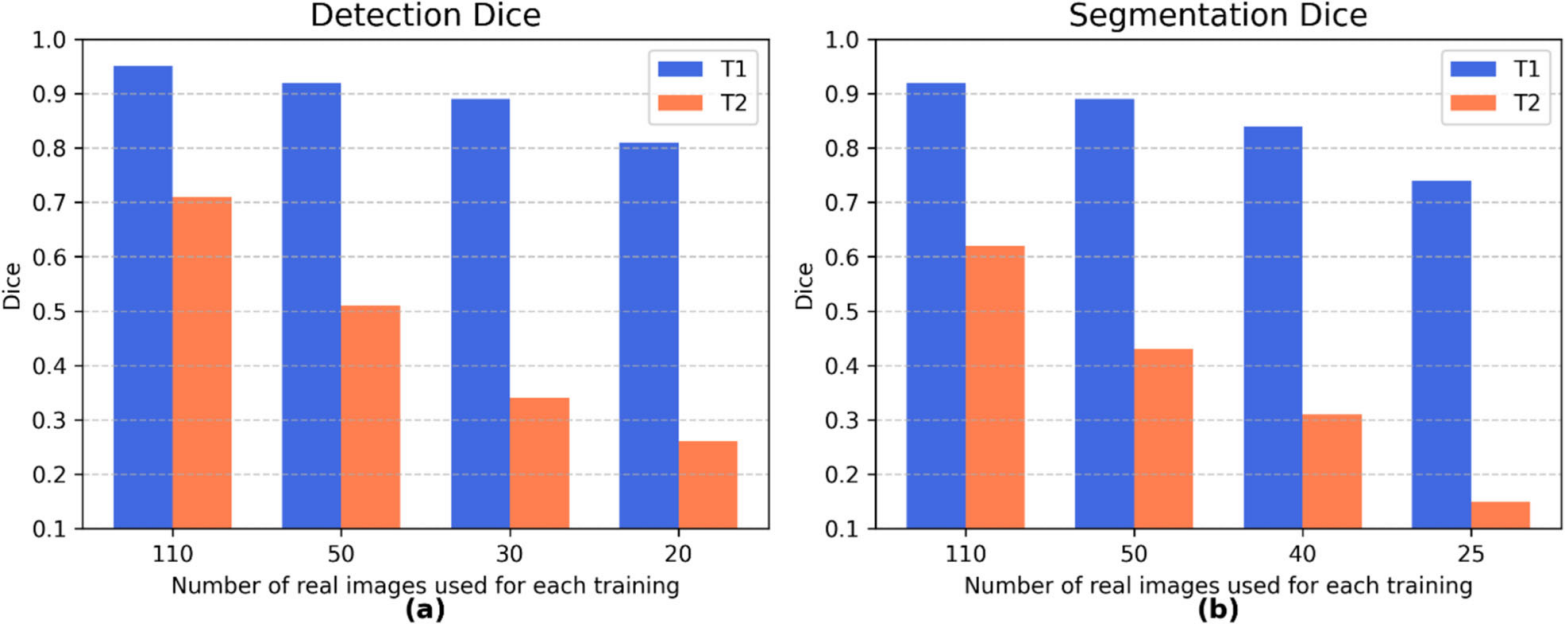
Performance evaluation, in both T1 and T2 real test sets, of detection (**a**) and segmentation (**b**) models using Dice score as a function of the decreasing number of real images included in the training, maintaining the same amount and type of synthetic images. For segmentation, smaller decrements in training images were used due to lower baseline performance and a steeper drop in Dice scores

The influence of real data quantity on model performance was evaluated on both T1 and T2 real test sets (Fig. 7). For detection, model performance on T1 dropped with as few as 20 training real images (Dice ∼ 0.8), but good detection performance can be maintained with just 30 real images. On the more challenging real test set (T2), detection Dice scores confirmed the trend but with lower absolute values. For segmentation, T1 testing showed high and stable Dice scores with 50 or more images, T2 highlighted a steeper drop in performance as the amount of real data decreased. This underscores the higher sensitivity of segmentation to real data quantity and the challenges of generalizing to unfamiliar domains.

## Discussion

This study presents a novel framework for generating synthetic datasets and employing hybrid training strategies to advance computer vision models for robotic suturing automation. The results demonstrated the potential of using synthetic datasets in advancing task-specific CV models while highlighting areas for further refinement to bridge the sim-to-real gap. A key achievement of this work lies in the development of a simple and reproducible framework for dataset generation and model training, utilizing Shapr3D, Unity, and the Perception package.

This pipeline facilitated the efficient creation of annotated, task-specific datasets, significantly reducing the manual annotation burden. By leveraging Unity’s automated annotation capabilities and incorporating randomization to enhance variability, this approach has proven effective in training CV models with good generalization capabilities. The study achieved real-time robust model performance with as few as 30–50 real images, underscoring the potential of hybrid training strategies to minimize reliance on real-world data.

The analysis of results revealed distinct trends in detection and segmentation tasks. In detection, increasing the realism of synthetic datasets led to progressively better performance on real-world data. However, even with enhanced realism, synthetic-only models were insufficient for complete generalization to real-world scenarios. Hybrid training, which combined synthetic and a minimal set of real images, proved necessary in overcoming this limitation, with Model 4 achieving a Dice score of 0.954 in real test data. Considering segmentation, the inherently higher complexity of the task resulted in lower performance for synthetic-only models even with increased realism. Nevertheless, the hybrid-trained Model 4 demonstrated a significant leap in performance, achieving a Dice Score of 0.920 with minimal reliance on real data. Notably, it also outperformed Model 3 across all synthetic test sets, demonstrating the benefits of hybrid training even within simulated contexts. Moreover, evaluation on an out-of-distribution real test set (T2) showed a performance drop compared to T1, with partial segmentation accuracy and reduced stability in real-time use; surgical tools and needle were generally well segmented, while cut and tissue remained the most challenging. This emphasizes the complexity of segmentation tasks and the need for more diverse data to ensure robustness across varying conditions.

While this study successfully addressed several research gaps, such as the scarcity of publicly available task-specific datasets and the minimization of manual data annotation efforts, limitations remain. Though improved, the realism of the synthetic datasets was insufficient to train models capable of fully generalizing to real-world contexts.

However, integrating advanced techniques such as generative adversarial networks (GANs), diffusion models, and normalizing flow models could further close the sim-to-real gap. These models have the potential to enhance the realism of synthetic datasets by refining visual characteristics and aligning them more closely with real-world images, thereby improving model generalization in real-world applications [17].

In addition to dataset realism, the study focused on type-based segmentation to classify and segment entire tools like the Cadiere Forceps and Needle Driver. However, future work must advance to part-based segmentation to identify individual components of tools, such as the arm, shaft, and clasper, where instance-based segmentation is even more relevant, being able to distinguish differently between objects belonging to the same class. This granularity is essential for tasks like pose estimation and action recognition, which are crucial for automating robotic suturing.

The hybrid instance segmentation model developed in this study provides a strong foundation for these advanced applications, supporting future efforts to integrate cognitive vision into surgical automation.

Moreover, emerging simulation platforms, such as PhysX-5, could also play a transformative role in advancing this research by offering simulation capabilities for soft tissue dynamics. Indeed, recent studies [18, 19] have demonstrated its utility in non-rigid and contact-rich surgical manipulations, suggesting its potential to generate datasets that incorporate realistic tissue interactions and deformations.

## Conclusion

This study contributes to robotic-assisted surgery by developing a comprehensive, task-specific framework for advancing cognitive vision and suturing automation. Indeed, key achievements include the creation of well-annotated, instance-based datasets tailored to robotic suturing, modifying, and designing 3D models specific to this domain and developing Unity environments for generating synthetic images with increasing realism. These resources not only address the scarcity of task-specific datasets but also provide researchers with reproducible and accessible tools to further explore this field.

Moreover, the hybrid training approach introduced in this study successfully minimized reliance on real data, achieving optimal model performance with as few as 30–50 real images. This method yielded a real-time instance segmentation model capable of bridging the gap between synthetic and real environments. With further improvements, this model will play a pivotal role in advancing pose estimation and surgical action recognition, essential steps toward full automation of robotic suturing. Furthermore, the publicly shared resources, including datasets, 3D models, and Unity environments with Randomizers, are designed to facilitate the fine-tuning of existing computer vision models, support semi-supervised learning approaches, and catalyze further progress in this domain. These contributions form the foundation for collaborative research efforts, emphasizing the importance of reproducibility and accessibility in fostering innovation.

Ultimately, this study lays the groundwork for iterative improvements in suturing automation and highlights the importance of cognitive vision in achieving precise and efficient surgical outcomes. It underscores the transformative role of publicly accessible resources in addressing critical research gaps, encouraging collaboration, and accelerating progress toward the goal of fully automated robotic suturing.

## Footnotes

1 3D Da Vinci Tools: https://github.com/jhu-dvrk/dvrk_cad/tree/main/Classic/PSM_CAD_Model/Tools.

2 *3D modeling software: *https://www.shapr3d.com.

3 *Unity Perception Package: *https://github.com/Unity-Technologies/com.unity.perception.

4 *Roboflow: *https://roboflow.com.

5 *Ultralytics repository: *https://github.com/ultralytics/ultralytics.

6 https://github.com/PietroLeoncini/Surgical-Synthetic-Data-Generation-and-Segmentation.

## Declarations

### Supplementary information

This article is accompanied by a supplementary video demonstrating the p erformance of the instance segmentation model developed in this study, providing visual evidence of the results discussed.
